# Granular Assembly of α-Synuclein Leading to the Accelerated Amyloid Fibril Formation with Shear Stress

**DOI:** 10.1371/journal.pone.0004177

**Published:** 2009-01-12

**Authors:** Ghibom Bhak, Jung-Ho Lee, Ji-Sook Hahn, Seung R. Paik

**Affiliations:** School of Chemical and Biological Engineering, College of Engineering, Seoul National University, Gwanak-Ku, Seoul, Korea; Swiss Federal Institute of Technology Lausanne, Switzerland

## Abstract

α-Synuclein participates in the Lewy body formation of Parkinson's disease. Elucidation of the underlying molecular mechanism of the amyloid fibril formation is crucial not only to develop a controlling strategy toward the disease, but also to apply the protein fibrils for future biotechnology. Discernable homogeneous granules of α-synuclein composed of approximately 11 monomers in average were isolated in the middle of a lag phase during the *in vitro* fibrillation process. They were demonstrated to experience almost instantaneous fibrillation during a single 12-min centrifugal membrane-filtration at 14,000×g. The granular assembly leading to the drastically accelerated fibril formation was demonstrated to be a result of the physical influence of shear force imposed on the preformed granular structures by either centrifugal filtration or rheometer. Structural rearrangement of the preformed oligomomeric structures is attributable for the suprastructure formation in which the granules act as a growing unit for the fibril formation. To parallel the prevailing notion of nucleation-dependent amyloidosis, we propose a double-concerted fibrillation model as one of the mechanisms to explain the *in vitro* fibrillation of α-synuclein, in which two consecutive concerted associations of monomers and subsequent oligomeric granular species are responsible for the eventual amyloid fibril formation.

## Introduction

Amyloidosis, a phenomenon in which soluble and innocuous proteins turn into amyloid fibrils through the highly specific self-assembly process, has been involved in various neurodegenerative disorders such as Parkinson's disease (PD), Alzheimer's disease (AD), and Prion disease [1], [2]. Mechanism of the suprastructure formation challenges us to an eventual development of controlling strategies toward the amyloidosis-related disorders [3] as well as applications of the protein fibrils in future nanobiotechnology [4], [5]. The emergence of ordering phenomenon out of a less-ordered or disordered state would also attract general interests in the areas of physical and materials sciences. It has been suggested that a principle for one type of fibril formation could be applied universally to various amyloid fibrils since those fibrils share the common final structure of cross β-sheet conformation [6], [7] even though the mechanism of fibrillation process is yet to be unequivocally unveiled.

α-Synuclein is a pathological component of PD by participating in the Lewy body formation as the major constituent [8], [9]. Increased level of this so-called ‘natively unfolded’ protein was closely related to the onset of PD [10], [11]. Triplication of the chromosome containing the α-synuclein gene caused the disorder [12]. Overexpression of the protein in transgenic animals such as mouse and *Drosophila* exhibited motor impairments reminiscent of human PD although the intracellular filamentous inclusions were found only in the flies with selective degeneration of dopaminergic neurons [13]–[15]. The identity of toxic species has been debated between the oligomeric protofibrils and the final amyloids [16]–[18]. Nevertheless, elucidation of the molecular mechanism of the amyloid fibril formation could provide us theoretical foundation to critically assess the neurodegenerative disorder for future development of therapeutic strategies [3].

As a widely recognized mechanism of amyloid fibril formation, the nucleation-dependent fibrillation suggests that the nucleation centers act as a template to accrete monomeric soluble protein by directing a specific conformational transformation for the production of insoluble protein fibrils [1], [19]–[21]. In this report, however, we present evidences to propose another mechanism of the fibrillation process in which the oligomeric granular species of α-synuclein turn into the amyloid fibrils through concerted lateral association of the preformed granules by inducing structural distortion of the preformed structures with a physical influence of shear stress.

## Results

### Granular structures of α-synuclein and their conversion to amyloid fibrils with centrifugal filtration

**Figure 1:**
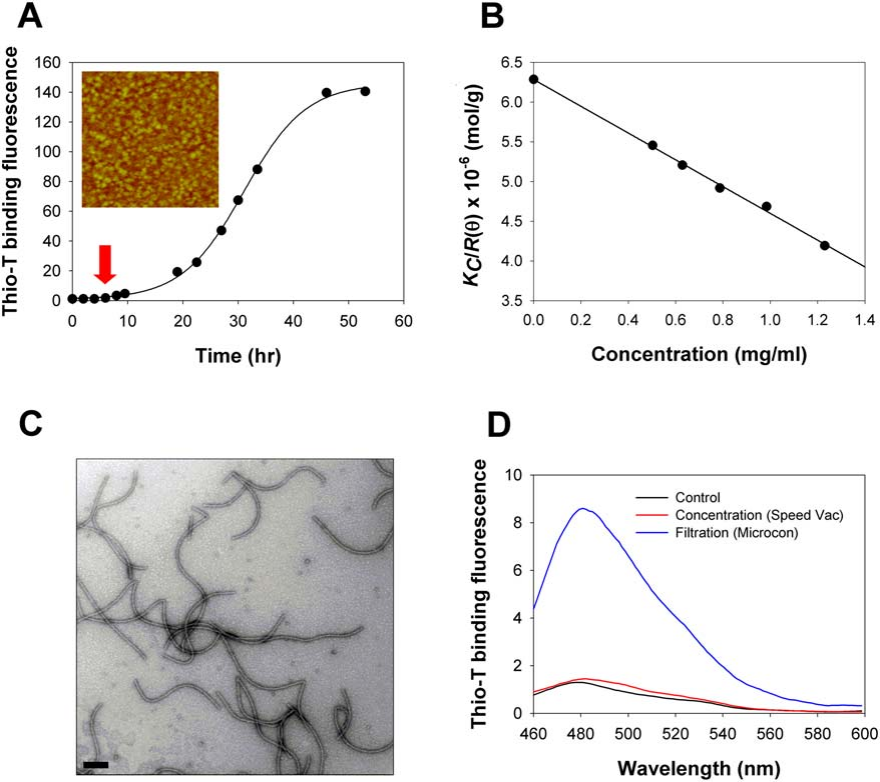
Oligomeric granules of α-synuclein and their conversion to amyloid fibrils. (A) Kinetics of amyloid fibril formation assessed with thioflavin-T binding fluorescence. α-Synuclein (1 mg/ml) in 20 mM Mes (pH 6.5) was incubated at 37°C with a continuous shaking. The oligomeric granules were obtained in the middle of the lag phase of the aggregation kinetics at 6 hour time-point (indicated by arrow). Microscopic image (2.5 µm×2.5 µm) of the granular structures obtained with AFM is provided (inset). (B) Debye plot of Rayleigh equation for the analysis of SLS data. The α-synuclein granules prepared in various concentrations (*C* = 0.504–1.23 mg/ml) were subjected to SLS spectrophotometric analysis with Otsuka Electronics DLS-7000 (k = 488 nm). The *KC*/*R_θ_* values for the granules were collected, and used to draw the Debye plot. (C) Instantaneous amyloid fibril formation of α-synuclein through the centrifugal filtration of the granules. The granules of α-synuclein (1 mg/ml) were subjected to a single centrifugal filtration at 14,000×g for 12 min at 37°C using Microcon YM-30 (Millipore). The fibrils were negatively stained with uranyl acetate on a copper grid (200 mesh), and examined with an EF-TEM. The scale bar represents 0.2 µm. (D) Failure of the fibril formation of the granules by the evaporation procedure using the speed vac lyophilization. The granular preparation (1 mg/ml) was subjected to dehydration and concentration with the speed vac lyophilizer for 3 hours (red) and single centrifugal filtration at 14,000×g for 12 min with Microcon YM-30 (blue) in comparison with the granules without any treatment (black). Following resuspension of the filtered and the concentrated preparations with 20 mM Mes to reach the initial 1 mg/ml concentration, thioflavin-T binding fluorescence spectra of the resulting products were monitored at between 460 nm and 600 nm with an excitation at 450 nm.

Granular forms of α-synuclein were collected during the fibrillation process of soluble α-synuclein incubated at 1 mg/ml in 20 mM Mes, pH 6.5, under a continuous shaking condition at 37°C. The granules were obtained in the middle of the lag period after 6 hours of the incubation by observing the granular homogeneity and their degree of population with microscopic images of either atomic force microscope (AFM) or transmission electron microscope (TEM) (Figure 1A). The granular preparation appeared to contain a negligible amount of monomeric α-synuclein, which was assessed by estimating the monomer content in the filtrate obtained via a brief centrifugal filtration of the granular preparation for 30 sec at 14,000×g with a large pore membrane (molecular weight cutoff = 100 kDa) (Figure S1). Even though the granules were distinctively monitored with dynamic light scattering (Figure S2), the granules were failed to be isolated intact via size-exclusion chromatography since those structures were converted back to the monomers (Figure S3). This fact indicates that the granules might exist in a pseudo-stable state although it remains to be clarified in future studies. Average diameter of the granules was determined as 18.9±2.6 nm from the TEM image. Average mass was estimated as 159 kDa from a Rayleigh equation applied to the static light scattering (SLS) data [22] (Figure 1B). By considering calculated molecular weight of monomeric α-synuclein as 14.4 kDa, one granule was determined to be assembled with approximately 11 molecules of α-synuclein. In addition, the negative *A_2_* value of −8.41×10^−7^ of the Debye plot (Figure 1B) indicated that these granules would be self-associative [22], [23].

In fact, the granules turned into the amyloid fibrils almost instantaneously by the centrifugal filtration procedure employing Microcon membrane filters (Figure 1C). A single centrifugal filtration for 12 min at 14,000×g was sufficient to produce the amyloid fibrils on the filter with molecular weight cutoff (MWCO) of 30 kDa. Under a normal shaking condition, those fibrils would be isolated only after a sufficient incubation for more than 50 hours of the entire fibrillation process (Figure 1A). Possible involvement of dehydration and/or concentration effects on the granules during the filtration-induced fibrillation was assessed with the centrifugal concentrator of speed vac lyophilizer (Ecospin 3180C, Hanil). The resulting concentrated granular preparation was examined for the fibrillar content with thioflavin-T binding fluorescence. While the single filtration gave rise to the maximal binding fluorescence at 482 nm, the speed vac concentrated granular preparation did not increase the thioflavin-T binding fluorescence from that of the pure granules (Figure 1D). This fact clearly indicates that neither dehydration nor concentration effect is responsible for the drastically accelerated fibrillation observed with the centrifugal filtration.

### Filtration-dependent amyloid fibril formation of α-synuclein

**Figure 2:**
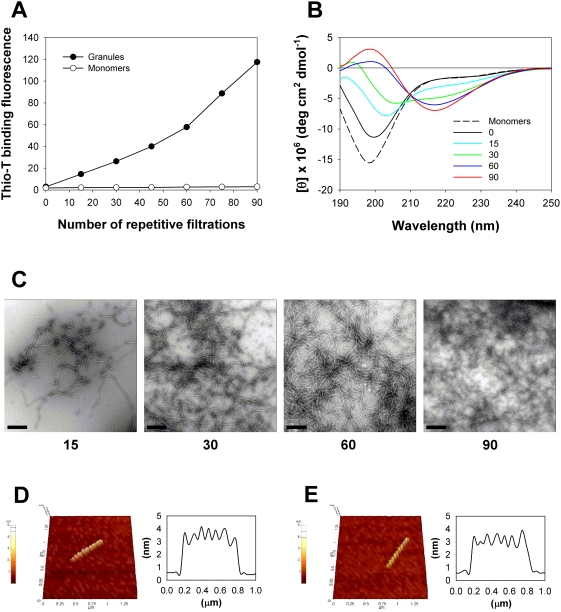
Filtration-dependent amyloid fibril formation of the α-synuclein granules. (A) Fibrillation of α-synuclein proportional to the number of repetitive filtrations. α-Synuclein in either granular (•) or monomeric (○) form was subjected to the repetitive centrifugal filtrations at 14,000×g with a 2-min cycle for each filtration at 37°C using Microcon YM-30 and YM-10, respectively. (B) Monitoring of the granules of α-synuclein with CD spectroscopy during the repetitive filtrations. The CD spectra were obtained for the granular preparation experiencing the centrifugal filtration following 0 (black), 15 (cyan), 30 (green), 60 (blue), and 90 times (red) of the repetition. The CD spectrum of monomeric α-synuclein is also shown in a dash line for comparison. (C) EF-TEM images of the amyloid fibrils obtained from the granules of α-synuclein following 15, 30, 60, and 90 times of the centrifugal filtrations with the 2-min cycle for each filtration. The scale bars represent 0.5 µm. (D) AFM 3D image (1.5 µm×1.5 µm) of single amyloid fibril obtained from the centrifugal filtration (left) and a line profile of the fibril (right). (E) AFM image (1.5 µm×1.5 µm) of a fibril produced by a normal shaking incubation (left) and its corresponding line profile (right).

The amount of amyloid fibrils as determined with thioflavin-T binding fluorescence increased proportionally with the number of centrifugal filtrations of the granules (Figure 2A). Following each 2-min filtration with Microcon YM-30, each filtrate was recombined with the original granular preparation to maintain the initial volume. Monomeric α-synuclein, on the other hand, did not convert into the fibrils even with 90 times of repetitive filtrations using Microcon YM-10. The enhanced amyloid fibril formation was also confirmed with the increased β-sheet content in the CD spectra of the α-synuclein granular preparation during the repetitive filtrations. The granules still existed mainly in random structure with the minimum ellipticity at 197 nm although its level was raised to some extent from the monomeric α-synuclein known to exist in a disordered state [24]–[26]. As the repetitive filtrations continued, the β-sheet content was gradually increased as the ellipticity at 220 nm became decreased, which was apparently changed from the initial random state as the granules reached to the fully matured amyloid state (Figure 2B). The augmented fibrillation was also demonstrated with negative stains of the fibrils revealed under TEM in the presence of uranyl acetate. As the repetitive filtrations proceeded from 0 to 90 times, the amount of negatively stained fibrils was dramatically increased (Figure 2C). A filtration-induced fibril was examined with AFM and it appeared to have a saw-looking surface (Figure 2D). That rugged shape was also shared by an amyloid fibril derived via a normal prolonged shaking incubation of monomeric α-synuclein (Figure 2E).

### Shear stress-induced amyloid fibril formation

**Figure 3:**
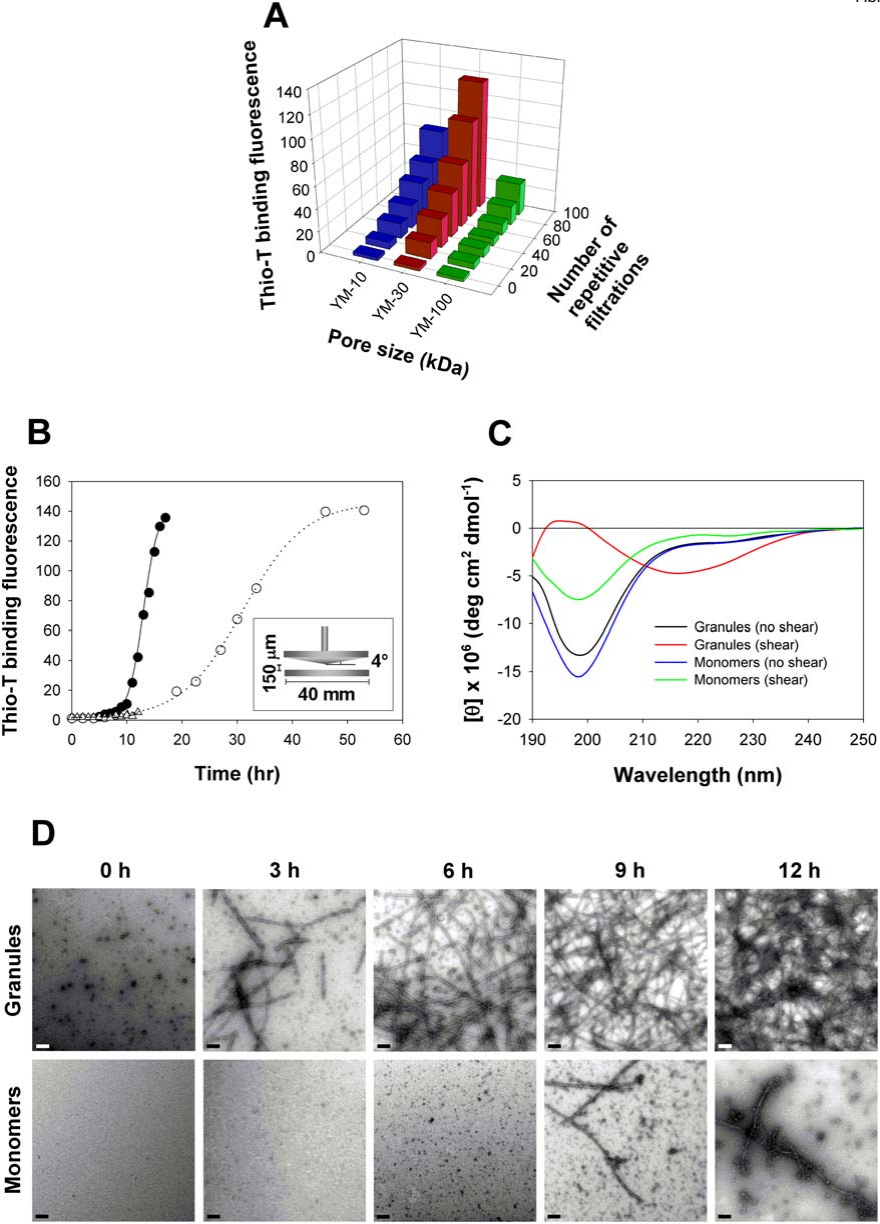
Shear-induced amyloid fibril formation of the α-synuclein granules. (A) Membrane pore size-dependence of the filtration-induced fibrillation of the granules. The repetitive centrifugal filtrations with the 2-min cycle per filtration were carried out at 14,000×g and 37°C for the granules of α-synuclein (1 mg/ml) with various centrifugal membrane filters of different pore sizes such as Microcon YM-10 (blue), YM-30 (red), and YM-100 (green). The extent of amyloid fibril formation was evaluated with the thioflavin-T binding fluorescence. (B) Fibrillation of the granules of α-synuclein under continuous shear stress imposed by a cone-and-plate type rheometer. Fibrillation kinetics of α-synuclein at 1 mg/ml in 20 mM Mes, pH 6.5, was monitored for either granular (•) or monomeric (△) state under 12 hours of continuous shear stress at 1 Pa. The kinetics is compared with the aggregation kinetics of monomeric α-synuclein under a normal shaking incubation at 200 rpm and 37°C (Figure 1A). (C) CD spectra of both granular and monomeric preparations of α-synuclein following 12 hours of the shear stress. The CD spectra are shown for the α-synuclein granules before (black) and after (red) the 12 hours of shear stress. For the monomers, their CD spectra were also obtained with (green) and without (blue) the shear stress. (D) The shear stress-induced amyloid fibrils were visualized as negatively stained EF-TEM images during the continuous shear stress applied to either granular or monomeric form of α-synuclein for various time intervals as indicated on the panels. All the scale bars represent 0.2 µm.

The filtration-induced fibrillation was dependent upon the pore size of the membrane filters. The repetitive centrifugal filtrations of the granular preparation were performed with a 2-min cycle for the filters of different pore sizes such as Microcon YM-10, YM-30, and YM-100 representing MWCO of 10 kDa, 30 kDa, and 100 kDa, respectively. During each 2-min cycle, the preparation (500 µl) was filtered to different amounts of 62.3±2.52 µl, 162.3±12.5 µl, and 349.3±3.06 µl depending on the pore sizes of the filters such as YM-10, YM-30, and YM-100, respectively. By recombining the filtrate with the original sample, the 2-min filtration cycle was continued. It turned out that Microcon YM-30 was the most effective membrane filter producing the fibrils in the greatest quantity while the other filters with wider (YM-100) or narrower (YM-10) pores did not produce the fibrils to that extent (Figure 3A). This pore size-dependent facilitated fibrillation suggests that the granule-based fibrillation might be due to a shear effect experienced by the preformed granular structures during the centrifugal filtration process. As a matter of fact, the occurrence of fluid shear during a membrane filtration process has been well recognized [27]. Proteins are shown to be affected by the filtration which would result in structural alterations of the proteins that lead to their subsequent aggregation and inactivation [28]–[33]. It was suggested to be due to the high shear stress generated by rapid solvent flow at the membrane surface. Amyloid fibril formation has also been suggested to be influenced by shear-inducing conditions such as stirring, shaking, mechanical agitation, and rheometric shear flow [34]–[37].

We have performed the shear-dependent amyloid fibril formation of the granular α-synuclein by employing a cone-and-plate type rheometer (C-VOR, Bohlin instruments) to impose the shear force to the protein in either granular or monomeric form. The granules were kept under continuous shear stress at 1 Pa by locating the granular preparation between a rotary top cone and a fixed bottom plate within the rheometer (Figure 3B, inset). Complete conversion of the granules to the amyloid fibrils was observed within less than 12 hours of shear stress whereas normal shaking-induced fibrillation required more than 50 hours (Figure 1A) to give rise to the same amount of the fibril formation as the shear-induced fibrillation (Figure 3B). In the case of the monomeric α-synuclein, however, the protein did not fibrillate as fast as the granular form. Instead, its fibrillation was no better than the amyloid formation induced by a normal shaking incubation. The reason why the pre-formed granules exerted much faster fibrillation compared with the fibrillation from monomers in the presence of the shear stress would be due to the altered physical status of the granular species induced with and without the shear force. In fact, careful examination of individual fibrils produced from those two types of granular species showed that the final fibrils appeared distinctive from each other in terms of their morphologies (Fig. 3D). The CD spectra to chase β-sheet formation also indicated that the predominant random structure of the granules has transformed into the β-sheet structure as the shear force was applied for 12 hours with the rheometer. The monomers, on the other hand, did not exhibit any significant level of β-sheet content even though the signal for random structure lowered to some extent (Figure 3C). The TEM images also showed that the extensive amyloid fibrils were drastically accumulated during the 12 hours of shear stress while the monomer hardly reached to the quantity of fibrils made from the granules (Figure 3D). Therefore, the filtration-induced fibrillation was proven to be caused by the shear force imposed on the granular structures of α-synuclein on the membrane filters. Moreover, it was also demonstrated that the fibril-producing shear stress was effective only on the preformed oligomeric granules, and not on the monomeric state of α-synuclein. In fact, there are several examples in which shear force has been considered to be a physical influence causing structural rearrangement in proteins. The oxy-form of sickle cell hemoglobin was described to be highly susceptible to the fluid shear forces [38]. A shear deactivation of cellulase enzyme complex was also reported [39]. Conformational change of von Willebrand factor, a protein present in the circulatory system, has been also demonstrated to be triggered by an increase in shear [40]–[43], which was directly visualized under shear flow using a microfluidic device [44].

### Granules as a growing unit for the amyloid fibril formation

**Figure 4:**
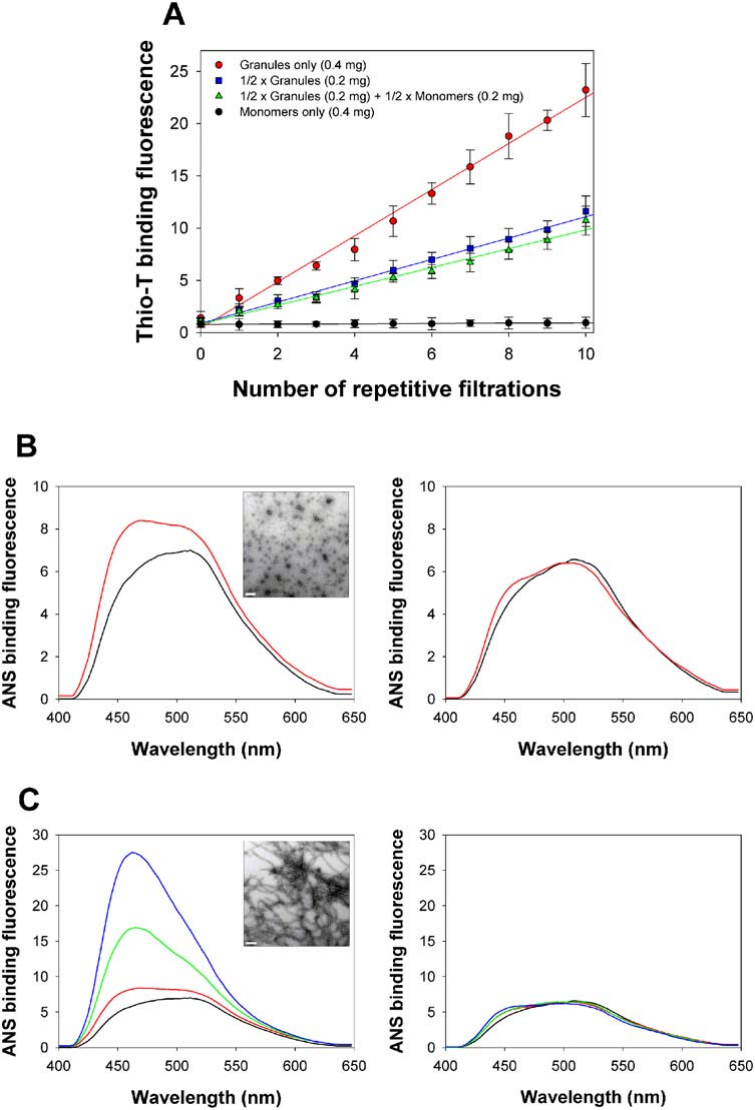
Granules as a growing unit for the amyloid fibril formation of α-synuclein. (A) Repetitive filtrations of the following α-synuclein preparations made of ‘Granules only (0.4 mg)’ (•), ‘1/2 Granules (0.2 mg)’ (▪), ‘1/2 Granules (0.2 mg)+1/2 Monomers (0.2 mg)’ (▴), and ‘Monomers only (0.4 mg)’ (•) at a total amount of 0.4 mg α-synuclein. The α-synuclein mixtures were repetitively centrifuged with Microcon YM-10 at 14,000×g and 37°C with a 5 min-cycle for each filtration. The fibril formation was monitored by the thioflavin-T binding fluorescence. The incremental slopes are 2.21 (•), 1.02 (▪), 0.90 (▴), and 0.015 (•). (B) ANS binding fluorescence spectra of the α-synuclein granules (left panel) and the monomers (right panel) before (black) and after (red) a single centrifugal filtration for 2 min with Microcon YM-10. An EF-TEM image of the α-synuclein granules after the filtration is shown (inset). The scale bar represents 0.2 µm. (C) The ANS binding fluorescence spectra of the granules (left panel) and the monomers (right panel) of α-synuclein were obtained after 15 (green) and 30 times (blue) of the repetitive filtrations with a 2-min cycle per filtration in addition to the spectra shown above for the α-synuclein preparations with (red) and without (black) the single filtration. The scale bar in the inset also represents 0.2 µm.

Since the monomers did not appear to be involved in the mature amyloid fibril formation during the filtration, only the granules were suspected to be sufficient to form the fibrils. This granule-only hypothesis was assessed with repetitive filtrations using Microcon YM-10. As the centrifugal filtrations were repeatedly applied to the granules with a 5-min cycle, their conversion to the fibrils, as monitored by the thioflavin-T binding fluorescence increased step-by-step with an average slope of 2.21 (Figure 4A). When the half of the granules were replaced with monomeric α-synuclein and subjected to the repetitive filtrations, the fibril formation became significantly suppressed to almost half of the original conversion efficiency with a slope of 1.02 (Figure 4A). This lowered conversion was certainly comparable with the fibrillation efficiency obtained when the remaining half of the granules was subjected to the filtrations in the absence of the monomers, which gave rise to another slope of 0.90 (Figure 4A). These facts clearly illustrate that the monomers hardly participate in the amyloid fibril formation of the granules during the repetitive filtration process. In other words, it is pertinent to consider that the amyloid fibrils of α-synuclein obtained during the filtration-induced fibrillation are produced only from the granules which act as a growing unit for the fibril formation. Depending on conditions of amyloidogenesis, however, α-synuclein and other α-synuclein-related molecules could also form amyloid fibrils via alternative mechanisms such as nucleation-dependent fibrillation.

To achieve the final suprastructure from the granular assembly, a structural rearrangement of the preformed granular structures was suspected to be responsible for the shear-induced filtration-dependent fibril formation [44], [45]. In fact, ANS binding fluorescence was monitored for both the monomers and the granules of α-synuclein during the repetitive filtrations of a 2-min cycle with Microcon YM-10. Even with a single filtration for 2 min, the ANS binding fluorescence of the granules was noticeably altered while that of the monomers was hardly affected (Figure 4B). The initial fluorescence spectrum with an emission maximum at 530 nm turned to a spectrum with a broad plateau that ranged between 460 nm and 520 nm (Figure 4B). Since the 2-min filtered materials did not contain any types of fibrils except the granules (Figure 4B inset) and their CD spectrum was not altered by the filtration, the blue shift of the maximum emission to 460 nm and the resulting broad fluorescence spectrum could be considered as an indication of the structural distortion of the granules experiencing the shear effect during the centrifugal filtration. When the number of the repetitive filtrations increased to 15 and 30 times during the entire incubations of 30 and 60 min, respectively, the emission maximum at 460 nm became prominent as the fibrils were produced from the distorted granules whereas the monomers were not affected by the increased number of filtrations (Figure 4C). Therefore, the structural distortion of the granular structures by the physical influence of shear stress, not by any sort of chemical influences, has been suggested to be responsible for the concerted assembly of the granules into the amyloid fibrils. Since the preformed oligomeric structures already contain the interactive interfaces in operation, the structural distortion of the preformed structures could rearrange the interactive surfaces within the granules, which may exchange their interactive counterparts to stabilize inter-granular interactions leading to the final suprastructure formation.

## Discussion

Emergence of a large structure from its constituting small pieces has been strenuously investigated in various disciplines. In general, molecular polymerization has been modeled in either stepwise or chain polymerization. For the amyloid fibril formation *in vitro*, a nucleation-dependent amyloidosis has been generally considered to be operated as the predominant mechanism, which is supported by the chain polymerization model [46], [47]. A thermodynamically unfavorable nucleus formation is followed by a rapid extension period reflecting accretion of monomers to the preexisting nucleation centers, which results in a characteristic sigmoidal aggregation kinetic curve with an initial lag period followed by accelerated fibril formation [48]. In this report, however, we present evidences to support the other model of stepwise polymerization for the amyloid fibril formation of α-synuclein. Our data indicate that the fibrils of α-synuclein are derived from dynamic association of the oligomeric granular structures which were previously made from the disordered monomeric α-synuclein in a concerted manner.

Formation of small oligomeric species at the earlier stages of the amyloid fibril formation has been demonstrated in several studies involving α-synuclein and Aβ [16], [49]–[52]. These oligomers convert into the β-sheet rich species from their relatively disordered states [1], [53]. In the nucleated conformational conversion (NCC) model, the flexible oligomers would experience structural rearrangement upon interaction with the nuclei, which eventually results in the amyloid fibril formation [54]. In the amyloidosis of yeast phosphoglycerate kinase, the oilgomers with critical mass have associated with one another to assemble into rather short protofibrils [55]. Recent study with all-atom computer simulation of the oligomerization process of Aβ peptides suggests that molten oligomers initially formed by sequestering hydrophobic residues from the solvent undergo a process of structural reorganization to form the extensive β-sheet fibrils [56].

**Figure 5:**
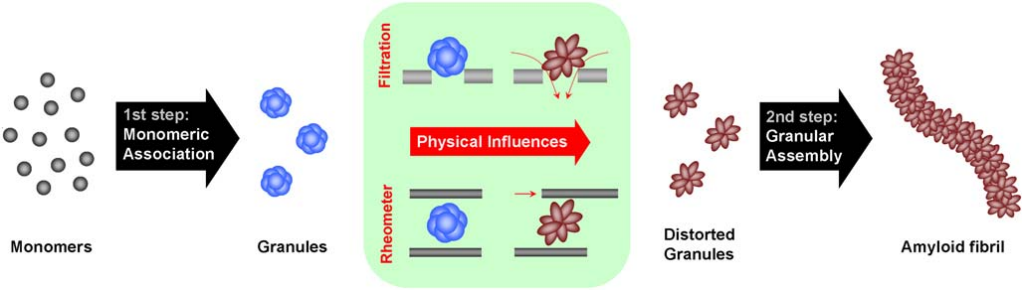
Schematic representation of the double-concerted fibrillation model of α-synuclein.

Based on the data we obtained, a novel double-concerted fibrillation model has been proposed to explain the granule-based fibrillation phenomenon, which would parallel the existing model of nucleation-dependent amyloid fibril formation. In our model, the granules occur through a concerted action of the monomeric proteins, which should be followed by another round of concerted process among the preformed oligomeric granules through the structural rearrangement imposed by a physical influence of the shear force (Figure 5). Therefore, we suggest that characteristic amyloid fibril formation could result from multiple pathways of molecular assembly that depend on amyloidogenic proteins/peptides; an alternative to the suggested generic mechanistic feature of the amyloid formation [1]. Nevertheless, the granular assembly leading to the amyloid fibril formation of α-synuclein introduced in this study is certainly one of the multiple mechanisms for the amyloid fibrillation process.

This investigation also led us to uncover a new mechanism of the amyloid fibril formation of α-synuclein identifying the preformed granular structures as a growing unit. Along with our previous finding that the granular species also turned into amyloid fibrils instantaneously in the presence of hexane [57], the structural distortion of the preformed oligomeric species is demonstrated to be a prerequisite for the amyloid fibril formation. The structural rearrangement of the preformed structure could occur inside the cell under various occasions. For instance, intracellular lipid membranes would provide an amphipathic interface where those structures could align to form the suprastructure. In this respect, various nanosurfaces of intracellular ultrastructures such as cytoskeletons could be also effective to alter the preformed species. In addition, various natural ligands of α-synuclein are also expected to influence the preformed structures. Transition of the granular species into the amyloid fibrils inside the cell may have a pathological significance. Since those granules have been suggested to be a toxic cause for the neuronal degeneration by affecting membrane stability [16], a possible conversion of the granules of α-synuclein into the amyloid fibrils on the lipid membrane surface could serve for a detoxification mechanism. As a matter of fact, we actually performed a cell study with human dopaminergic neuroblastoma cells (SH-SY5Y) to evaluate the toxic effect of the granular species and the filtration-induced fibrils of α-synuclein at 0.1 mg/ml. The cell survival was significantly affected by the granules with % survival of 55.2% while the amyloid fibrils increased the survival to 87.6%, indicating that the fibrils became less toxic than the granules. Alternatively, however, the on-going process of surface-dependent suprastructure formation could also affect the cell viability by providing physical influences on the membrane structures. Cellular effects of those materials, therefore, await future investigation.

In addition, the biochemical significance of the granular structures of α-synuclein needs to be also emphasized in terms of their crucial roles on the amyloidogenesis. Those granular structures would be more functional during the amyloid fibril formation beyond their dominantly suggested roles as either simple intermediates of fibrillation or even off-pathway products [16], [58]. Besides the biomedical significance, the assembly of the preformed granular structures should also contribute to not only our understanding of fundamentals of suprastructure formation but also producing protein-based nanomaterials for applications in the area of future biotechnology.

## Materials and Methods

### Preparation of α-synuclein

Recombinant α-synuclein was prepared according to the procedures previously described [59]. Briefly, α-synuclein cloned in pRK172 was overexpressed in *E. coli* BL21(DE3) and completely purified via heat treatment of the cell lysate, DEAE-Sephacel anion-exchange, Sephacryl S-200 size-exclusion, and S-Sepharose cation-exchange chromatography steps. Purified α-synuclein was stored in aliquots at −80°C following dialysis against 6 liters of 20 mM Mes at pH 6.5.

### Preparation of α-synuclein granules and thioflavin-T binding fluorescence

To obtain oligomeric granules of α-synuclein, kinetics of amyloid fibril formation were monitored with thio-T binding fluorescence. α-Synuclein (1 mg/ml) in 20 mM Mes (pH 6.5) was incubated at 37°C with a continuous shaking on an orbit shaker at 200 rpm. Aliquots (20 µl) of α-synuclein (1 mg/ml) were combined with 2.5 µM thioflavin-T in 50 mM glycine, pH 8.5, to a final volume of 200 µl. Amyloid formation was evaluated with thioflavin-T binding fluorescence at 482 nm with an excitation at 450 nm. The fluorescence intensity was measured with a luminescence spectrometer (LS-55, Perkin-Elmer). α-Synuclein granules were obtained in the middle of the lag phase of the aggregation kinetics.

### Static light scattering (SLS) spectrophotometer

The oligomeric granules of α-synuclein prepared in various concentrations (*C* = 0.504–1.23 mg/ml) were subjected to SLS spectrophotometric analysis with Otsuka Electronics DLS-7000 (k = 488 nm). The *KC*/*R_θ_* values for the granules were collected, and used to draw the Debye plot. For the analysis of SLS data to obtain the molecular mass, the following Rayleigh equation has been employed:
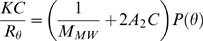
(1)where *R_θ_* represents the Rayleigh ratio between scattered vs. incident lights, *C* is the solute concentration in g/ml, *M_MW_* is the average molecular weight, and *A_2_* is 2^nd^ virial coefficient in mol/g^2^. The *K* term is an optical constant as defined in
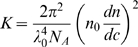
(2)where *N_A_* is Avogadro's constant, *λ*_0_ is laser wavelength, *n*_0_ is solvent refractive index, and *dn*/*dc* is the differential refractive index increment (*dn*/*dc* = 0.14187 in this study). The *P*(*θ*) is angular dependence of the scattered light intensity. If the particles in solution are far smaller than the wavelength of the incident light, the *P*(*θ*) term is reduced to 1, which has been known as Rayleigh scattering. Then, the Rayleigh scattering can be expressed in
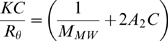
(3)which is used to draw the Debye plot between *KC*/*R_θ_* and *C* on y- and x-axis, respectively. The average molecular weight (*M_MW_*) is determined from the y-intercept and the 2^nd^ virial coefficient (*A_2_*) is obtained from the slope.

### Centrifugal membrane filtration of the α-synuclein granules

A single centrifugal membrane filtration of α-synuclein granules (1 mg/ml) was performed at 14,000×g for 12 min at 37°C using Microcon YM-30 (Millipore), which filtered an initial volume (500 µl) of the granular preparation in 20 mM Mes to a final 10 µl. The resulting amyloid fibrils were collected via a reverse centrifugation after resuspending the filtered preparation with the same initial volume of 20 mM Mes, pH 6.5. For the repetitive centrifugal filtrations, α-synuclein in either granular or monomeric state was subjected to the filtrations at 14,000×g with a 2-min cycle for each filtration at 37°C using Microcon YM-30 and YM-10, respectively. Following each filtration for 2 min, the filtrate was combined to the original sample, and subjected to another round of the filtration. As the filtration was repeated for 500 µl of the α-synuclein preparation in 20 mM Mes, pH 6.5, at 1 mg/ml, aliquots of the repetitively filtered materials were analyzed. Membranes of the Microcon filters are made of regenerated cellulose exerting the low-binding, anisotropic, and hydrophilic properties.

### 8-anilino-1-naphthalenesulfonic acid (ANS) binding fluorescence

α-Synuclein (35.7 µM) and ANS (25 µM) were combined in 20 mM Mes, pH 6.5, and subjected to a single centrifugal filtration for 2 min with Microcon YM-10. Following resuspension of the filtered preparations with the filtrate (20 mM Mes) to reach the initial concentration, the resulting products were collected via a reverse centrifugation. The ANS binding fluorescence was monitored with emission scan between 400 and 650 nm following an excitation at 350 nm. Fluorescence of ANS was measured with a luminescence spectrometer (LS-55, Perkin-Elmer).

### Cone-and-plate type rheometer

The effect of shear stress on fibrillation of α-synuclein was investigated with a cone-and-plate type rheometer (C-VOR, Bohlin instruments). After α-synuclein in either granular or monomeric form at 1 mg/ml in 20 mM Mes, pH 6.5, was placed between a rotary top cone and a fixed bottom plate within the rheometer, continuous shear stress at 1 Pa was imposed to the protein with the rheometer at 37°C for 12 hr. To monitor fibrillation kinetics of α-synuclein, aliquots (25 µl) were collected at one hour interval and assessed with the thioflavin-T binding fluorescence.

### Energy-filtering transmission electron microscope (EF-TEM)

The amyloid fibrils were visualized with EF-TEM (LIBRA 120, Carl Zeiss). An aliquot (5 µl) containing the protein aggregates of α-synuclein was adsorbed onto a carbon-coated copper grid (200 mesh) and air-dried for 2 min. After negative staining with 2% uranyl acetate (Electron Microscopy Sciences) for another 30 sec, the aggregates were examined with EF-TEM.

### Atomic force microscope (AFM)

Aliquot (5 µl) containing granules or amyloids of α-synuclein were placed on freshly cleaved mica. Following adsorption of the protein aggregates, the droplet was displaced with 100 µl of distillated water. After removal of excess water, the samples were analyzed with Dimension 3100 AFM (Figure 1A) and XE-100 AFM (Figure 2D and E) in tapping mode.

### Circular dichroism (CD) spectroscopy

Secondary structures of granules and amyloids were monitored with CD spectroscopy (J-715, Jasco) in 20 mM Mes at pH 6.5. The spectra were measured within a 0.1-mm pathlength quartz cell between 195 and 250 nm with a step resolution of 1.0 nm, bandwidth of 1.0 nm, and scan speed of 20 nm/min. All of the spectra were obtained from an average of five scans.

### Cell culture

Human dopaminergic neuroblastoma cells (SH-SY5Y) were grown in DMEM supplemented with 10% fetal bovine serum in 5% CO_2_ at 37°C. The cells were plated in a 96-well plate at 5.0×10^4^ cells/well. After the cell growth reached to 80–90% confluence, the granules and the amyloid fibrils of α-synuclein were separately added to the cells at 0.1 mg/ml, and further incubated for 20 hours in 5% CO_2_ at 37°C. The live cells were directly counted with and without trypan blue staining by observing them with an inverted microscope.

## Supporting Information

Figure S1 Monomer content within the granular preparation. α-Synuclein (1 mg/ml) was subjected to a brief centrifugal filtration at 14,000×g for 30 sec at 25°C using Microcon YM-100. Monomers collected in the filtrates were monitored with BCA assay. The monomers recovered from the samples prepared with only monomers at 1 mg/ml and 1∶1 mixture of monomers and granules at 0.5 mg/ml each were recovered as expected via the brief centrifugation (first and second bar). Our granular preparation (1 mg/ml), however, was shown to hardly contain any monomers (third bar).
(0.73 MB TIF)

Figure S2 Dynamic light scattering of monomers and granules of α-synuclein. Hydrodynamic radius of the granular (•) species of α-synuclein was evaluated with dynamic light scattering (DLS) measurement at 25°C in comparison with the monomeric form (○) using Photal dynamic laser scattering spectrometer DLS-7000 (Otsuka Electronics Co.).
(0.65 MB TIF)

Figure S3 Size-exclusion chromatography of the granular species. (A) The elution profiles of size-exclusion chromatography for either granular (•) or monomeric (○) preparations of α-synuclein (1 mg/ml). Each sample was applied onto Sephacryl 200 HR column (10 mm×175 mm). The chromatography was carried out with 20 mM Mes, pH 6.5 at a flow rate of 0.4 ml/min. (B) SDS-PAGE and (C) Native-PAGE of the monomeric and granular forms of α-synuclein are also shown.
(1.39 MB TIF)
