# Supplementary figures and images for: Granular Assembly of α-Synuclein Leading to the Accelerated Amyloid Fibril Formation with Shear Stress
Source: PLoS One. 2009 Jan 12;4(1):e4177. doi: 10.1371/journal.pone.0004177 (PMC2613562; doi:10.1371/journal.pone.0004177)

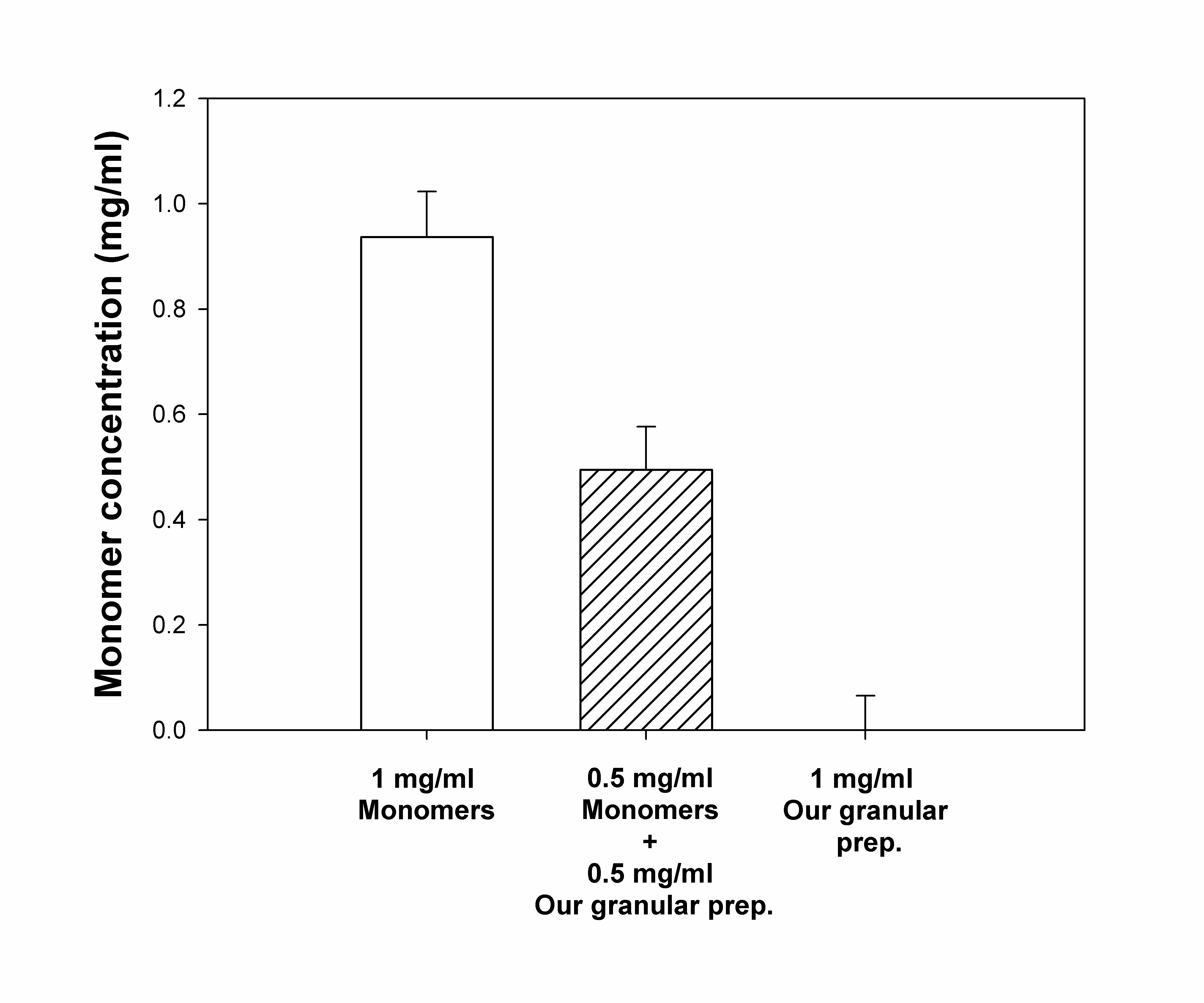

Supplement: Figure S1 — Monomer content within the granular preparation. α-Synuclein (1 mg/ml) was subjected to a brief centrifugal filtration at 14,000×g for 30 sec at 25°C using Microcon YM-100. Monomers collected in the filtrates were monitored with BCA assay. The monomers recovered from the samples prepared with only monomers at 1 mg/ml and 1∶1 mixture of monomers and granules at 0.5 mg/ml each were recovered as expected via the brief centrifugation (first and second bar). Our granular preparation (1 mg/ml), however, was shown to hardly contain any monomers (third bar). (0.73 MB TIF) [file pone.0004177.s001.tif]

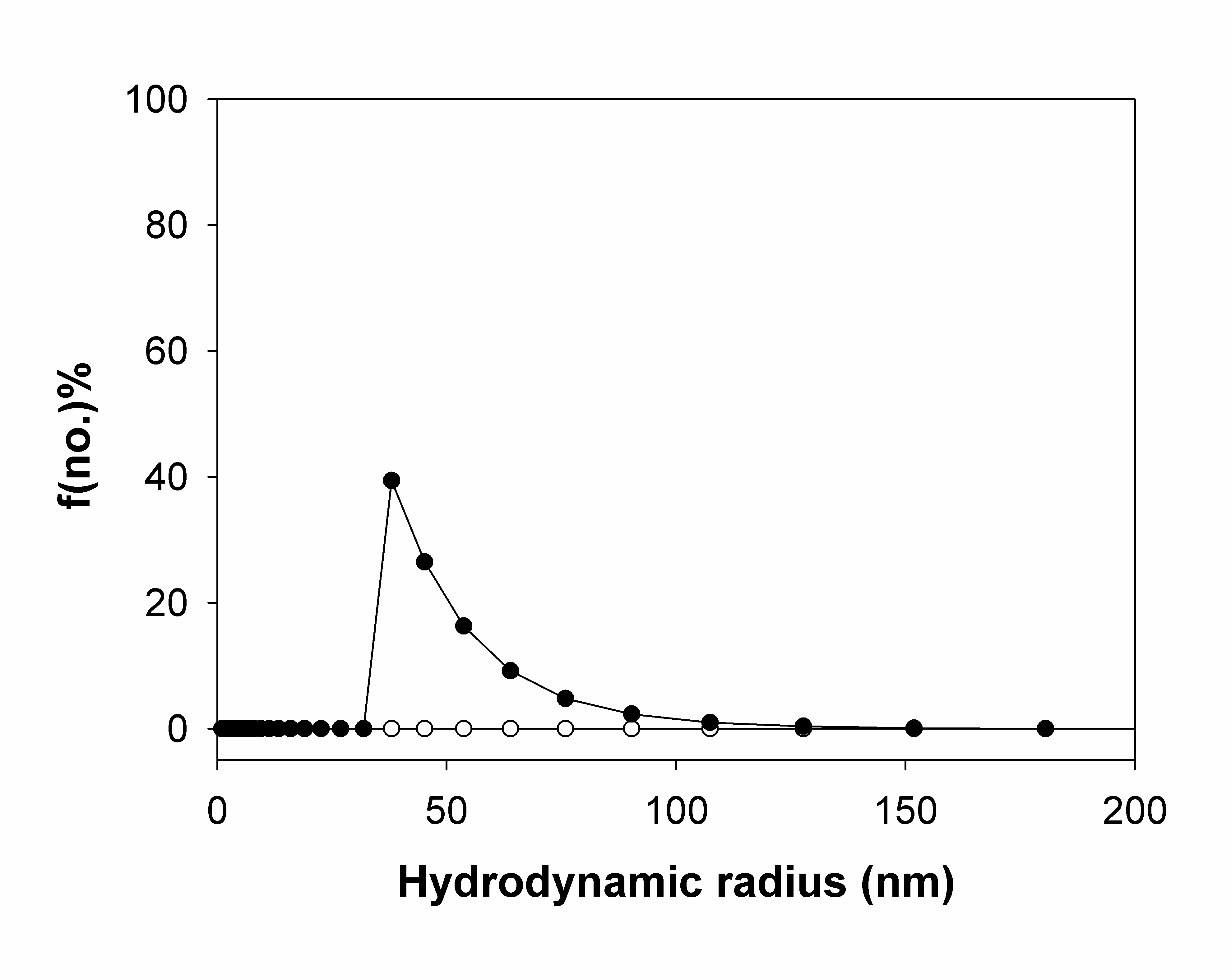

Supplement: Figure S2 — Dynamic light scattering of monomers and granules of α-synuclein. Hydrodynamic radius of the granular (•) species of α-synuclein was evaluated with dynamic light scattering (DLS) measurement at 25°C in comparison with the monomeric form (○) using Photal dynamic laser scattering spectrometer DLS-7000 (Otsuka Electronics Co.). (0.65 MB TIF) [file pone.0004177.s002.tif]

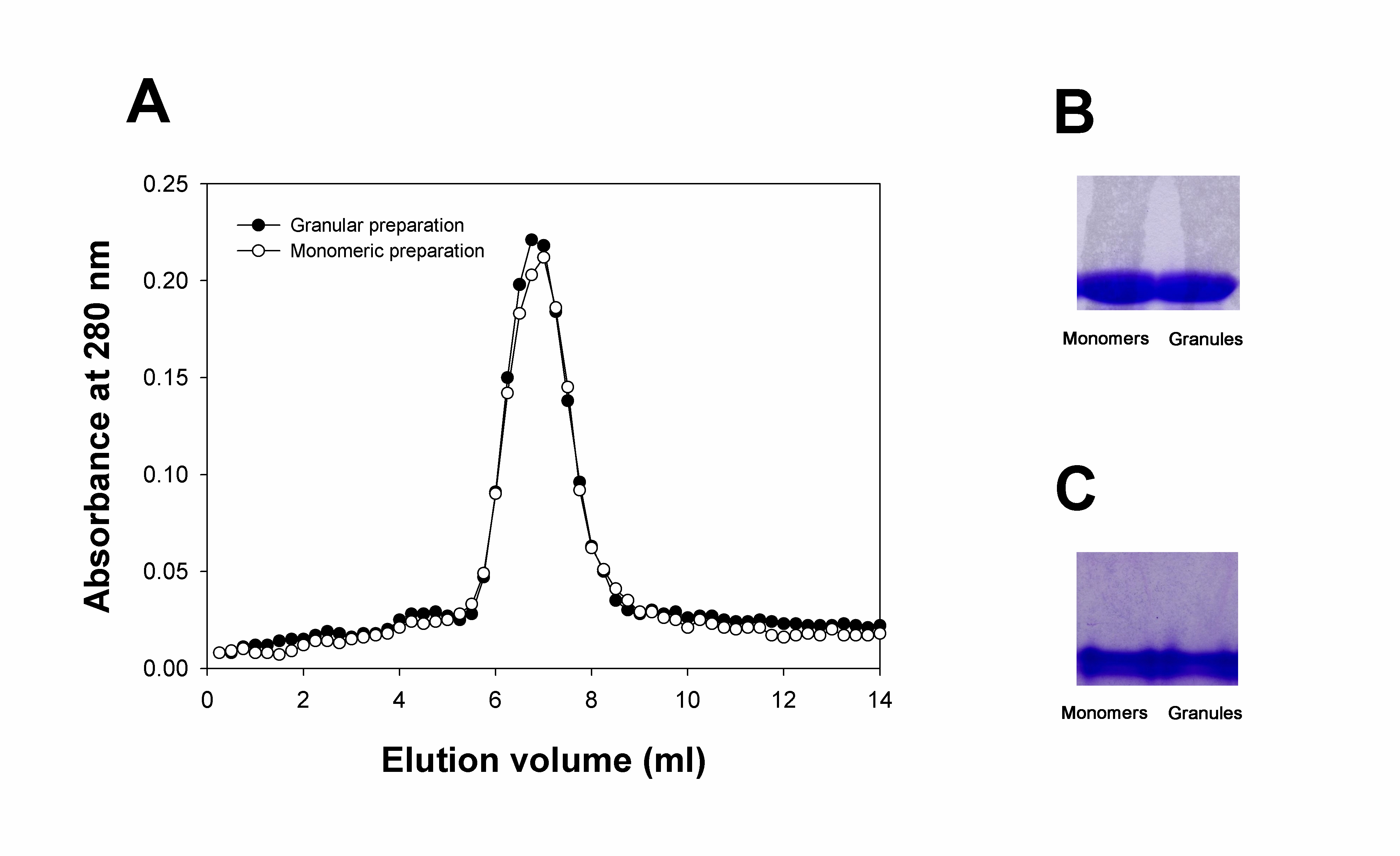

Supplement: Figure S3 — Size-exclusion chromatography of the granular species. (A) The elution profiles of size-exclusion chromatography for either granular (•) or monomeric (○) preparations of α-synuclein (1 mg/ml). Each sample was applied onto Sephacryl 200 HR column (10 mm×175 mm). The chromatography was carried out with 20 mM Mes, pH 6.5 at a flow rate of 0.4 ml/min. (B) SDS-PAGE and (C) Native-PAGE of the monomeric and granular forms of α-synuclein are also shown. (1.39 MB TIF) [file pone.0004177.s003.tif]
